# Management of pregnancy and delivery in women with Kawasaki disease and residual coronary artery lesion with detailed analysis of labor analgesia: combined experience of 13 cases in two institutions

**DOI:** 10.1186/s40981-020-00375-y

**Published:** 2020-09-07

**Authors:** Rie Inoue, Yusuke Mazda, Hiroaki Tanaka, Kayo Tanaka, Jun Yoshimatsu, Kazumi Tamura, Katsuo Terui

**Affiliations:** 1 Department of Anesthesiology and Pain Medicine, Juntendo University, 3-1-1 Hongo, Bunkyo-ku, Tokyo, 113-8431 Japan; 2 Department of Anesthesia and Pain Management, Mount Sinai Hospital, University of Toronto, Toronto, Canada; 3 Department of Obstetrics and Gynecology, Mie University Faculty of Medicine, Tokyo, Mie Japan; 4 Department of Perinatology and Gynecology, National Cerebral and Cardiovascular Center, Osaka, Japan; 5 Division of Obstetric Anesthesia, Department of Anesthesiology, Saitama Medical Center, Saitama Medical University, Saitama, Japan

**Keywords:** Kawasaki disease, Coronary artery disease, Pregnancy, Delivery, Neuraxial analgesia

## Abstract

**Introduction:**

Approximately half of Kawasaki disease patients are expected to have transitioned to adulthood, and an increasing number of patients with cardiovascular sequelae have gotten pregnant. Management of women with Kawasaki disease who have residual coronary artery disease is poorly established. Thus, we conducted detailed analysis of these cases.

**Methods:**

We reviewed 19 pregnancies in 13 such women in two tertiary perinatal facilities, Saitama Medical Center and National Cardiovascular Center. The medical records were reviewed in all women with Kawasaki disease and coronary artery lesion between 1998 and 2015, with regard to age of diagnosis, types of coronary artery lesion, location, previous treatment, pregnancy course and medical management for coronary lesion, cardiac function, and planned mode of delivery.

**Results:**

Fourteen parturients attempted vaginal delivery, and all but one received neuraxial analgesia, providing stable hemodynamics. Four elective and two emergency cesarean deliveries were performed due to obstetric indications, while one woman required cesarean delivery at preterm due to maternal cardiac indication.

Among 14 attempted vaginal deliveries, instrumental vaginal delivery was performed in 50%. Cardiac events were noted in four women, all in post-partum period, such as non-sustained ventricular tachycardia or chest discomfort without ECG changes. Antithrombotic medication was exclusively low dose aspirin in 11 of 19 pregnancies (58%), and none received anticoagulation during pregnancy or delivery.

**Conclusion:**

Our case series support the practice of preferred vaginal delivery, with neuraxial labor analgesia in indicated patients, while highlighting the need for vigilance in the post-partum period.

## Introduction

Since the first report of Kawasaki disease in 1967 [1], the mortality has dramatically decreased to 4 in 100,000 in a recent report [2]. As the incidence remains high not only in Japan but also in the USA [3], many women with residual coronary artery lesion are now reaching childbearing age, and the number is estimated approximately 120,000 worldwide [4]. However, only two relatively large case series are reported to guide the obstetrical care and management of this patient population [5, 6]. Tsuda et al. reported 46 deliveries in 30 women by nationwide survey in Japan [5]. Gordon et al. reported 21 pregnancies in 10 women by single institution registry [6]. These studies report excellent outcome with no cardiac event during parturition and suggest that cesarean delivery can be reserved for obstetrical indications.

When managing these women with residual coronary artery lesion, it is important to alleviate hemodynamic stress during labor and immediate post-partum period. Tachycardia may provoke myocardial ischemia in patients with stenotic lesion, and arterial wall stress with pain-related hypertension may result in coronary artery aneurysm rupture. Epidural analgesia is known to effectively block sympathetic nervous system activation during labor and delivery, and it is actually provided in one third of patients in the report by Tsuda [5]. Unfortunately, it is not clear from her study that epidural analgesia during labor was effective, because detailed information of labor analgesia and hemodynamics during labor are lacking. The other report by Gordon does not describe analgesia during labor at all [6]. Thus, we aimed to investigate whether labor epidural analgesia is effective in stabilizing hemodynamics during parturition by reviewing patients with coronary artery lesion in two high-volume centers in Japan.

## Methods

After IRB approval of Saitama Medical Center (#1010) and National Cerebral and Cardiovascular Center (M25-026), medical records were reviewed in all women with Kawasaki disease and coronary artery lesion between 1998 and 2015, with regard to age of diagnosis, coronary artery lesion with its type, location, and previous treatment, pregnancy course and medical management for coronary lesion, cardiac function, and planned mode of delivery. We waived to obtain written consent from each individual due to retrospective manner, which was approved by the IRBs.

Detailed review of labor epidural analgesia was also conducted with regard to the method of neuraxial analgesia, anesthetic agents, monitoring during labor, presence or absence of bearing down for delivery, duration of labor, mode of delivery, and any cardiac event or hemodynamic changes. Neonatal outcomes were also reviewed by gestational age at delivery, Apgar scores, and umbilical artery pH.

## Results

A total of 13 women with 19 pregnancies met the inclusion criteria. Nine women were managed in National Cerebral and Cardiovascular Center, and 4 were managed in Saitama Medical Center. The ages of the patients were between 18 and 36 years old. Eight of them were nulliparous, and five were parous. Table 1 showed the details of coronary artery lesion, medication during pregnancy, mode of delivery, and cardiovascular events during and after delivery.

**Table 1:** Patient cardiac status

Case ID	Age	NYHA	RCA lesion	LCA lesion	LV function	Medication	Cardiovascular event
1	28	I	Occlusion (#1:100%)	Aneurysm (large)	EF 40%	LDA	Decreased EF to 30% upon delivery, NSVT on POD#5
2	18	I	Aneurysm	Localized stenosis Aneurysm (large)	EF 63%	None	No
3 (#1)	31	I	Occlusion (grafted)	Aneurysm (large, grafted)	EF 50%	LDA	No
3 (#2)	33	I			EF 59%		No
4 (#1)	34	I	Aneurysm (large)	Localized stenosis (stented)	EF 64%	LDA	No
4 (#2)	36	I			EF 57%		No
5 (#1)	29	I	Aneurysm (large)	Aneurysm	EF 80%	None	No
5 (#2)	32	I			EF 80%		No
6	34	I	Localized stenosis (grafted)	SVG to LAD	WNL	None	Chest discomfort without ECG and TTE changes, diuretic given for oliguria
7 (#1)	25	II	Localized stenosis (#2:70%, #3:50%) Aneurysm	Localized stenosis (#6:50%) Aneurysm	FS 40%	LDA	Chest discomfort, O2 supplementation
7 (#2)	29	II			FS 40%		No
8	27	I	Localized stenosis Aneurysm	Aneurysm	WNL	None	No
9 (#1)	28	I	Localized stenosis Aneurysm	Localized stenosis Aneurysm	WNL	LDA	No
9 (#2)	31	I			WNL		No
10 (#1)	24	I	None	Aneurysm	WNL	LDA	No
10 (#2)	26	I			WNL	None	No
11	34	I	Localized stenosis	Localized stenosis	WNL	ISDN	No
12	36	II	Localized stenosis	Localized stenosis	EF 60%	LDA	Tachyarrhythmia required lidocaine and carbedilol on POD#1, NSVT on POD#13
13	31	I	Aneurysm	Aneurysm (large)	EF 59%	None	No

*NYHA* New York Heart Association, *RCA* right coronary artery, *LCA* left coronary artery, *LV* left ventricle, *EF* ejection fraction, *FS* fractional shortening, *LDA* low-dose aspirin, *CD* cesarean delivery, *NSVT* non-sustained ventricular tachycardia, *SVG* saphenous vein graft, *LAD* left anterior descending, *WNL* within normal limit, *TTE* transthroracic echocardiogram, *ISDN* isosorbide dinitrate

Right coronary artery (RCA) lesion was present in 12/13 patients (92%), left sided lesion was present in 100% of patients. Types of coronary artery lesion were as follows. Both aneurysm and stenosis: 6(46%), aneurysm only: 3(23%), stenosis only: 3(23%), and dissection only: 1(7.6%). Three patients had undergone coronary artery interventions before pregnancy, i.e., coronary artery bypass graft: 2, coronary stent placement: 1. One patient had echocardiographic and ECG evidence of old myocardial infarction (patient #1), and only one of the patients had anginal symptoms, which necessitated early termination of pregnancy at 33 weeks gestation by cesarean delivery.

Antithrombotic medication was exclusively low dose aspirin in 11 of 19 pregnancies (58%), and none received anticoagulation during pregnancy or delivery. One patient was on daily nitrate, and none was taking βblocker.

Cardiac function was fairly good by NYHA classification (I: 16/19 pregnancies, II: 3/19, III: 0/19). Two women with NYHA class II were those who became class II during pregnancy. One of them underwent cesarean delivery at preterm due to frequent chest discomfort (patient #7), and the other underwent cesarean delivery at 35 weeks due to non-sustained ventricular tachycardia (patient #12) on Holter ECG. Ejection fraction was equal to or greater than 40% in all cases during pregnancy, but one patient who had EF of 40% during pregnancy showed decrease of EF from pre-pregnancy value of 60% (patient #1).

Details of anesthesia management were shown in Table 2. Elective cesarean delivery was performed in 5 pregnancies with indication of previous C/S in 2 (patient #7-2 and #10-2), breech presentation in 1 (patient #13), maternal arrhythmia in 1 (patient #12), and maternal chest discomfort in 1 (patient #7-1). Emergency cesarean section was performed in two patients (patient #1 and #10-1), both due to failure to progress.

**Table 2:** Anesthetic and obstetric details

**Vaginal delivery**					
Case ID	Indication of neuraxial analgesia	Labor/mode	Technique	Monitoring	Neuraxial modality
2	Physician recommendation	Spontaneous NSVD	Epidural	ECG, NIBP, SpO2, IBP	CEI 0.1% Ropivacaine
3 (#1)	Physician recommendation	Spontaneous NSVD	Epidural	EGC, NIBP, SpO2	CEI 0.1% Ropivacaine
3 (#2)	Patient requirements	Spontaneous Forceps	Epidural	EGC, NIBP, SpO2	CEI0.1% Ropivacaine
4 (#1)	Physician recommendation	Spontaneous Forceps	Epidural	ECG, IBP	PIEB+PCEA 0.1% Ropivacaine
4 (#2)	Physician recommendation	Spontaneous NSVD	None	None	N/A
5 (#1)	Physician recommendation	Induced Forceps	Epidural	ECG, NIBP, SpO2	IEB, 1% Lidocaine and 0.25% Ropivacaine
5 (#2)	Physician recommendation	Induced Forceps	Epidural	ECG, NIBP, SpO2	IEB, 1% Lidocaine & 0.25% Ropivacaine
6	Physician recommendation	Spontaneous Forceps	Epidural	ECG, NIBP, SpO2, CVP(post-partum)	IEB, 1% Lidocaine
8	Physician recommendation	Spontaneous NSVD	Epidural	ECG, NIBP, SpO2, IBP, CVP	IEB, 1% Lidocaine
9 (#1)	Physician recommendation	Spontaneous Forceps	Epidural	ECG, NIBP, SpO2	CEI 0.1% Ropivacaine
9 (#2)	Physician recommendation	Induced vacuum	Epidural	ECG, NIBP, SpO2	CEI 0.1% Ropivacaine
11	Physician recommendation	Spontaneous Vacuum	Epidural	ECG, NIBP, SpO2	IEB, 1% Lidocaine
**Cesarean delivery**					
Case ID	Indication of cesarean delivery	Urgency	Anesthesia	Monitoring	Anesthetic management
1	Failure to progress	Urgent	Epidural	ECG, NIBP, SpO2, IBP, PAC, TTE	Dopamine infusion for hypotension
7 (#1)	Chest discomfort	Elective	General	ECG, NIBP, SpO2, IBP	Prophylactic ISDN
7 (#2)	Repeat	Elective	CSEA	ECG, NIBP, SpO2	Prophylactic ISDN
10 (#1)	Failure to progress	Urgent	CSEA	ECG, NIBP, SpO2	Ephedrine bolus for hypotension
10 (#2)	Repeat	Elective	CSEA	ECG, NIBP, SpO2	Ephedrine bolus for hypotension
12	NSVT	Elective	CSEA	ECG, NIBP, SpO2	Ephedrine and phenylephrine bolus for hypotension, lidocaine infusion
13	Breech presentation	Elective	CSEA	ECG, NIBP, SpO2	Ephedrine and phenylephrine bolus for hypotension, PDPH

*NSVD* normal spontaneous vaginal delivery, *NIBP* non-invasive blood pressure, *IBP* invasive blood pressure (arterial line), *CEI* continuous epidural infusion, *PIEB* programmed intermittent epidural bolus, *PCEA* patient control epidural analgesia, *IEB* intermittent manual epidural bolus, *CVP* central venous pressure, *PAC* pulmonary artery catheter, *TTE* transthracic echocardiogram, *ISDN* isosorbide dinitrate, *CSEA* combined spinal epidural anesthesia, *NSVT* non-sustained ventricular tachycardia, *PDPH* post-dural puncture headache

Among women attempting vaginal delivery, labor induction was planned in only 4 out of 14 pregnancies (29%). Neuraxial labor analgesia was provided in all but one pregnancy (93%). One woman received epidural analgesia for her first pregnancy, but did not have time to receive epidural analgesia in her second pregnancy due to rapid progress of labor (patient #4-2). The reasons for epidural analgesia were patient’s request in one, while in the rest of them neuraxial analgesia was recommended by obstetric and anesthetic care providers. Mode of delivery among these women with neuraxial analgesia was forceps delivery in 6(46%), normal spontaneous vaginal delivery in 3(23%), emergency cesarean delivery in 2(15%), and vacuum extraction in 2(15%).

Intrapartum hemodynamic monitoring was routine, i.e., ECG, non-invasive blood pressure monitoring, and pulse oximetry in 6 out of 14 attempting vaginal deliveries (43%). Only arterial line was added to routine monitors in one case, while both central line and arterial line was placed in two cases. Pulmonary artery catheter (PAC) was inserted in one patient (patient #1) who developed worsening ejection fraction from pre-pregnancy 64 to 40% during pregnancy. In this patient, echocardiogram showed severe hypokinesis in posterior wall and hypokinesis in anteroseptal region. Previous coronary angiography (CAG) showed 100% occlusion in #1 with good collateral flow, 50% stenosis in #7. Persantine thallium test at 35 weeks gestation showed redistribution in both RCA and left anterior descending (LAD) regions. Thus, we expected that PAC revealed the possible effect of autotransfusion during uterine contraction as well as upon delivery of the infant, while aiding the diagnosis of acute myocardial ischemia.

The method of neuraxial labor analgesia was either continuous epidural infusion (CEI) with or without patient controlled epidural analgesia (PCEA) at Saitama Medical Center, while intermittent epidural bolus was used at National Cerebral and Cardiovascular Center. The concentration of local anesthetic was more dilute in the former, i.e., 0.08 to 0.1% ropivacaine with 0.0002% fentanyl. In the latter, 1% lidocaine combined with 0.25% bupivacaine was used without fentanyl. Anesthesia-related complications include post-dural puncture headache (PDPH) in 1 patient, while in 4 out of 14 pregnancies (29%), these patients experienced brief episodes of hypotension after epidural analgesia, which was easily treated either by ephedrine or phenylephrine bolus. Contrary to the neuraxial analgesia during vaginal delivery, complications during cesarean delivery tended to be severe. Five out of 7 cesarean deliveries (5 elective and 2 emergency) required vasopressor for hypotension during epidural anesthesia.

**Fig. 1:**
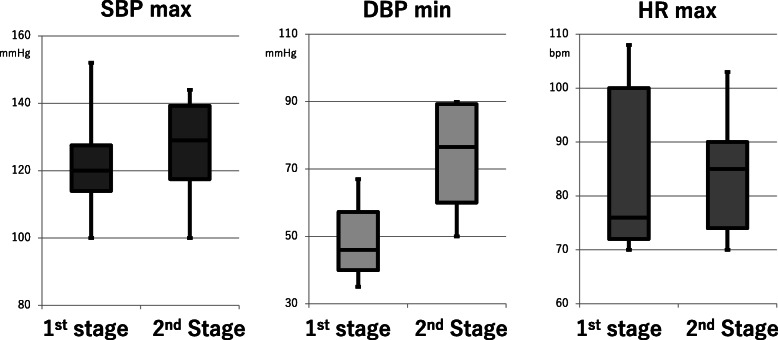
Hemodynamic parameters during labor and delivery under anesthesia. Legends: SBP; systolic blood pressure, DBP: diastolic blood pressure, HR: heart rate

Figure 1 showed the blood pressure and heart rate during labor and delivery with epidural analgesia. Highest systolic blood pressure (SBP) of all women was 152 mmHg, while lowest diastolic BP was 35 mmHg. Highest HR was 103 bpm, documenting favorable myocardial oxygen demand/supply ratio with neuraxial labor analgesia.

Table 3 demonstrated the neonatal outcomes. Four out of 19 cases (21%) were delivered preterm. Three of them were delivered by semi-elective cesarean delivery due to maternal condition, while the other case was vaginal delivery due to preterm labor at 23 weeks gestation. Neonatal conditions were generally good as evidenced by umbilical artery pH greater than 7.0 in all cases. Apgar scores were also good except the neonate delivered at 23 weeks gestation. There was no evidence that either neuraxial labor analgesia or maternal coronary lesion adversely affected neonates.

**Table 3:** Neonatal outcome

Case ID	Gestational week at delivery	APGAR 1 min	APGAR 5 min	Delivery	UApH	Neonatal condition
1	38	8	9	Cesarean	7.319	Healthy
2	38	9	9	Vaginal	7.408	Healthy
3 (#1)	40	8	8	Vaginal	7.159	Healthy
3 (#2)	39	9	9	Vaginal	7.324	Healthy
4 (#1)	38	8	8	Vaginal	7.236	Healthy
4 (#2)	40	8	8	Vaginal	7.275	Healthy
5 (#1)	39	7	9	Vaginal	7.213	O2 support
5 (#2)	38	9	9	Vaginal	7.252	Healthy
6	38	9	9	Vaginal	7.449	Healthy
7 (#1)	33	6	7	Cesarean	Not recorded	Not recorded
7 (#2)	34	9	9	Cesarean	7.359	Healthy
8	23	6	6	Vaginal	7.294	NICU admission
9 (#1)	38	9	10	Vaginal	Not recorded	Healthy
9 (#2)	37	8	8	Vaginal	Not recorded	Healthy
10 (#1)	39	8	8	Cesarean	Not recorded	Healthy
10 (#2)	38	8	9	Cesarean	7.282	Healthy
11	40	8	9	Vaginal	7.288	Healthy
12	35	8	9	Cesarean	7.361	O2 support
13	37	8	9	Cesarean	Not recorded	Healthy

*UApH* umbilical arterial pH, *NICU* neonatal intensive care unit

Cardiac event or hemodynamic changes occurred in 4 women. One patient developed hypotension (SBP 80 mmHg) after delivery of the infant and oxytocin administration during cesarean delivery (case #1). Transthoracic echocardiogram revealed decreased EF to 30% from pre-delivery value of 40%. On suspicion of autotransfusion upon delivery and/or negative inotropic effect of oxytocin, dopamine was administered for 30 min until recovery of EF to 45%. This patient also developed non-sustained ventricular tachycardia on POD#2 upon first ambulation attempt. She was started on coronary dilators post-operatively. CAG on POD#15 showed similar findings compared to pre-pregnancy CAG. Two other women complained of chest discomfort after delivery after returning to ward, but without the evidence of myocardial ischemia. They were treated with oxygen supplementation in one, and diuretic administration for oliguria in the other. The last woman developed recurrent tachyarrhythmia on POD#1, for which lidocaine and carvedilol dosages were increased. She developed non-sustained ventricular tachycardia on POD#13 and transferred to cardiology ward.

## Discussion

Our study confirmed the previous nationwide survey [5] and recent case series [6] in that most women with Kawasaki disease and coronary artery lesion tolerate pregnancy and vaginal delivery well, when pre-pregnancy cardiac function is good, and with proper medical management. Cesarean section can be reserved for obstetrical indications in most cases, but some may need preterm delivery due to maternal condition. Low-dose aspirin were prescribed in some, but none received anticoagulation during delivery. This practice prevented excessive post-partum hemorrhage in our case series. Thus, our current practice is in accordance with the most recent guidelines for diagnosis and management of cardiovascular sequelae in Kawasaki disease by Japanese Circulation Society, published in 2013 [7].

However, question remains as to the benefits and risks of neuraxial analgesia in these patients during delivery. Ischemic heart disease is considered to be a good indication of neuraxial labor analgesia, because it will relieve pain-mediated tachycardia and hypertension, while blocking sympathetic nervous system below 10th thoracic nerve. This vasodilatation is expected to alleviate hemodynamic effect of autotransfusion with each uterine contraction. Our case series showed stable hemodynamics during labor and vaginal delivery in these patients with significant coronary artery lesions. The most recent guidelines acknowledge the benefit of combined practice of neuraxial labor analgesia and instrumental vaginal delivery in order to avoid cardiac effects of bearing down effort [7]. However, in our case series, bearing down in the second stage of labor was allowed in 5 out of 6 deliveries where description of bearing down could be retrieved from the medical records. It is very likely that other five cases without record also pushed during delivery. It is our opinion that bearing down effort could be allowed based on baseline cardiac function as well as close hemodynamic assessment of its effect in each woman. Also, there seems to be no need to electively assist delivery by vacuum of forceps in the second stage.

With regard to the need for neuraxial analgesia during labor, there is no prospective randomized study in this small group of patients. Both Saitama Medical Center and National Cerebral and Cardiovascular Center can provide neuraxial labor analgesia on 24-h basis and believes in the theoretical benefit of neuraxial analgesia in certain cardiac lesions, including coronary artery stenosis. However, the nationwide survey in Japan by Tsuda showed that epidural analgesia was provided in only 33% of 27 vaginal deliveries. It is not clear from the survey that the epidural rate was higher when women with only stenotic lesions were included. Conducting randomized controlled trial to elucidate the benefit of neuraxial analgesia in this patient population would be extremely difficult. We can probably say that, as the authors of the survey suggest, woman with coronary artery lesion can expect uneventful pregnancy and delivery course if coronary artery lesion is not stenotic, and her cardiac function is normal prior to pregnancy. In women with significant coronary artery stenosis or symptomatic myocardial ischemia, we believe that combination of neuraxial analgesia, avoidance of pushing effort, and instrumental vaginal delivery to shorten the second stage of labor are beneficial to minimize hemodynamic event during labor and delivery.

The risk of cardiac events during labor in women with aneurysmal lesion from Kawasaki disease is another issue to be considered. There has been no report of aneurysm rupture during pregnancy or delivery [8]. However, aneurysm dilatation during pregnancy has been clinically observed. Also, pathological examination of coronary artery aneurysm in Kawasaki disease revealed endothelial damage, calcification, and narrowed diameter in some cases [9, 10]. There has been a case report of ventricular fibrillation and cardiac arrest during pregnancy in a woman with coronary artery aneurysm [11]. We think women with coronary artery aneurysmal lesion would also benefit from close evaluation during pregnancy and neuraxial analgesia during labor. We also acknowledge that neuraxial labor analgesia is not mandatory, as was the case in the second childbirth in case #4, whose labor progressed very rapidly.

The indication of invasive hemodynamic monitoring is another controversial issue in managing these women. Arterial line was inserted in 54% of cases who were attempting vaginal delivery. They were helpful in managing early recognition of hemodynamic derangements, especially in women with arrhythmia. Also, they aided in deciding whether bearing down could be repeated for delivery. However, we also noted that, in repeated pregnancy in the same patient, invasiveness of hemodynamic monitoring decreased. This may be due to the comfort in care providers from the successful management of their previous labor and delivery. With the development of less invasive or non-invasive continuous hemodynamic monitoring, the need for invasive monitoring in these women may be decreased in the future. Central line placement may be more for the administration of vasoactive drugs, rather than hemodynamic monitoring in this case series.

Cardiac events in this case series occurred in the post-partum period in all four cases, mostly ventricular arrhythmias. This observation supports the importance of careful post-partum observation with appropriate monitoring on the post-partum ward. One episode of hemodynamic compromise occurred immediately after delivery when auto-transfusion was most pronounced. It is important to recognize that this is the most dangerous period in women with cardiovascular disease.

Pre-pregnancy counseling in subsequent pregnancy warrants another important attention. We report 6 women with 2 pregnancies. None of them had progression of coronary artery lesions nor worsening NYHA classification. However, the disease progression in Kawasaki disease is quite variable, and some adults remain asymptomatic despite myocardial damage. Also, atherosclerotic lesion may overlap preexisting coronary artery lesions. According to the 2018 guidelines of the Japanese Circulation Society, at the time of the first consultation of pregnancy, sufficient medical history, family history, and current symptoms should be heard, consultation with a basic physician, and when a pregnancy is identified regardless of the severity of heart disease It is desirable to conduct early evaluation of the pregnancy follow-up including pre- and post-partum pregnancy follow-up by conducting an evaluation using cardiac ultrasound and electrocardiogram, and to provide prompt medical treatment according to the dynamic cardiovascular changes of the perinatal mother [12]. It is very important to prevent dropout of these women from follow-up and to accumulate experience of managing such women for labor and delivery by the multidisciplinary team of pediatric and adult cardiologist, obstetrician, midwives and nurses, and anesthesiologist to confer best outcome for both mothers and babies.

## Conclusion

This case series in two institutions support the recent guidelines in that most women with Kawasaki disease and coronary artery lesion tolerate pregnancy and vaginal delivery well when pre-pregnancy cardiac function was good, and with proper medical management. However, some may still require cesarean delivery at preterm due to maternal condition. Neuraxial labor analgesia was provided in most of the cases and stabilized hemodynamics. Indications for neuraxial analgesia and invasive hemodynamic monitoring should be individualized depending on the type and severity of coronary lesion and cardiac function.

## Data Availability

Not applicable
